# Editorial: Pediatric Microbiome in Health and Disease: Recent Advances

**DOI:** 10.3389/fped.2022.908741

**Published:** 2022-05-05

**Authors:** Amy Y. Pan, George M. Weinstock, Jill L. Maron

**Affiliations:** ^1^Division of Quantitative Health Sciences, Center for Microbiome Research, Department of Pediatrics, The Medical College of Wisconsin, Milwaukee, WI, United States; ^2^The Jackson Laboratory for Genomic Medicine, Farmington, CT, United States; ^3^Department of Pediatrics, Women and Infants Hospital of Rhode Island, Providence, RI, United States

**Keywords:** microbiome, preterm birth, feeding methods, type 1 diabetes, antibiotics, neurodevelopment

In the past decade, we have gained new knowledge regarding maternal microbiota transmission to a newborn, gut microbial colonization in early life, and the temporal progression of the composition of the gut microbiota during childhood development. We are just beginning to understand the significant role microbial colonization plays in the health and disease within the pediatric population. Maternal microbiota, mode of delivery, feeding methods, timing of the introduction to solid foods, nutrition, and antibiotics can influence the microbiome which then impacts heath and growth of a child. In addition, microbiome composition alterations may be involved in the pathogenesis of many diseases such as preterm birth, autoimmune disease, neurodevelopmental and neuropsychiatric disorders.

This month's Research Topic aims to highlight the latest research on the development of the microbiome in children and to elucidate the roles of the microbiome in health and disease states in the pediatric population. This comprehensive Research Topic, composed of five original article and comprehensive reviews, provides the reader with the most recent advances in the field and will serve as an invaluable resource for pediatric microbiome researchers.

In the observation study by Aguilar-Lopez et al., the impact of human vs. bovine nutritional fortification on the developing microbiome and weight gain velocity in preterm infants born <33 weeks of gestation or <1,500 grams at birth is described. The authors also explored the effects of maternal complications, including chorioamnionitis and preterm, prolonged rupture of membranes (PPROM) on fecal taxonomic composition. Compared to non-exposed infants, those exposed to chorioamnionitis during pregnancy had higher relative abundances of Bifidobacterium and Enterobacter, while infants born in the setting of PPROM showed higher abundances of Bacteroides. Samples clustered into two distinct groups when analyzing beta diversity which leads to characterization of two different enterotypes in these preterm infants.

Wang et al., advance our understanding of the impact of microbial colonization on the metabolome during the first 4 weeks of life. Through *in vitro* composition and volume assessment of intestinal gases and short-chain fatty acid (SCFA) profiles, the authors determine that total volume of intestinal gases as well as the concentration of carbon dioxide, hydrogen, methane, hydrogen sulfide, and all six fecal SCFAs analyzed are increased with age. Carbon dioxide and acetic acid were identified as the predominant intestinal gas and SCFA, respectively.

The mini review by Prescott et al. examines how intrapartum antibiotic prophylaxis (IAP) impacts infant microbial colonization. Decreased Bacteroidetes and Actinobacteria and increased Proteobacteria are seen after IAP, which might lead to alteration of normal pattern of colonization in infants. This review suggests that the effect size and duration of this alteration might be extended by cesarean section delivery and formula feeding.

Laue et al. review the association between microbiome and neurodevelopment with emphasis toward children from birth to 3 years of age, which is considered as a critical developmental window and interventions may be the most effective.

Rounding out the Research Topic update, Zhao et al. interrogate original data from multiple databases to investigate intestinal microbiota abundance from (a) healthy adults and children, (b) individuals with type 1 diabetes (T1D) across the age spectrum, and (c) individuals with T1D limited to children 0–18 years old. The relative abundance of Firmicutes was found to be higher in cases while that of Bacteroidetes was higher in healthy controls. The study also suggests that *Prevotella copri* has a high abundance in children with T1D from birth to 11 years old, while *Bacteroides vulgatus* has a high abundance in those across different age spectrums.

Combined, these articles provide novel insight into the diverse and long-lasting effect of the microbiome in health and disease states from preterm infants through to adolescents and serve as a valuable resource for researchers interested in the pediatric microbiome.

## Author Contributions

All authors listed have made a substantial, direct, and intellectual contribution to the work and approved it for publication.

## Conflict of Interest

The authors declare that the research was conducted in the absence of any commercial or financial relationships that could be construed as a potential conflict of interest.

## Publisher's Note

All claims expressed in this article are solely those of the authors and do not necessarily represent those of their affiliated organizations, or those of the publisher, the editors and the reviewers. Any product that may be evaluated in this article, or claim that may be made by its manufacturer, is not guaranteed or endorsed by the publisher.

